# Supplementary material for: All-at-once RNA folding with 3D motif prediction framed by evolutionary information
Source: Nat Methods. 2025 Oct 3;22(10):2094–106. doi: 10.1038/s41592-025-02833-w (PMC12510878; doi:10.1038/s41592-025-02833-w)
Supplement: Supplementary file 3 — Source code to run CaCoFold-R3D. Data and results. [file 41592_2025_2833_MOESM3_ESM.gz › supplemental_material/results/CaCoFold-R3D/CaCoFold-R3D_Rfam_vshuffle/RF02150_MESTIT1_3.R2R.sto.pdf]

RF02150_MESTIT1_3

5′-U G R G U R G A C ● U G U U C Y ● U G G C Y U U C A G A A U R A G A U C C U U U G U G R G C A C U C A G U C C A U U A A ● U A ● A A A A U U G C C U U C A U C U U G G A U G C U G G U G Y U U C U G R
